# Transcriptome analysis reveals the molecular mechanisms of the defense response to gray leaf spot disease in maize

**DOI:** 10.1186/s12864-018-5072-4

**Published:** 2018-10-11

**Authors:** Yang Yu, Jianyang Shi, Xiyang Li, Jian Liu, Qi Geng, Haichun Shi, Yongpei Ke, Qun Sun

**Affiliations:** 1 Key Laboratory of Bio-resources and Eco-environment of the Ministry of Education, College of Life Sciences, Sichuan University, Chengdu, People’s Republic of China; 2 Agronomy College, Sichuan Agriculture University, Chengdu, Sichuan People’s Republic of China

**Keywords:** Gray leaf spot (GLS), RNA-seq, WGCNA, Disease resistance, Maize, MRM

## Abstract

**Background:**

Gray leaf spot (GLS), which is caused by the necrotrophic fungi *Cercospora zeae-maydis* and *Cercospora zeina*, is one of the most impactful diseases in maize worldwide. The aim of the present study is to identify the resistance genes and understand the molecular mechanisms for GLS resistance.

**Results:**

Two cultivars, ‘Yayu889’ and ‘Zhenghong532,’ which are distinguished as resistant and susceptible cultivars, respectively, were challenged with the GLS disease and a RNA-seq experiment was conducted on infected plants at 81, 89, 91, and 93 days post planting (dap). Compared with the beginning stage at 81 dap, 4666, 1733, and 1166 differentially expressed genes (DEGs) were identified at 89, 91, and 93 dap, respectively, in ‘Yayu889,’ while relatively fewer, i.e., 4713, 881, and 722 DEGs, were identified in ‘Zhenghong532.’ Multiple pathways involved in the response of maize to GLS, including ‘response to salicylic acid,’ ‘protein phosphorylation,’ ‘oxidation-reduction process,’ and ‘carotenoid biosynthetic process,’ were enriched by combining differential expression analysis and Weighted Gene Co-expression Network Analysis (WGCNA). The expression of 12 candidate resistance proteins in these pathways were quantified by the multiple reaction monitoring (MRM) method. This approach identified two candidate resistance proteins, a calmodulin-like protein and a leucine-rich repeat receptor-like protein kinase with SNPs that were located in QTL regions for GLS resistance. Metabolic analysis showed that, compared with ‘Zhenghong532,’ the amount of salicylic acid (SA) and total carotenoids in ‘Yayu889’ increased, while peroxidase activity decreased during the early infection stages, suggesting that increased levels of SA, carotenoids, and reactive oxygen species (ROS) may enhance the defense response of ‘Yayu889’ to GLS.

**Conclusion:**

By combining transcriptome and proteome analyses with comparisons of resistance QTL regions, calmodulin-like protein and leucine-rich repeat receptor-like protein kinase were identified as candidate GLS resistance proteins. Moreover, we found that the metabolic pathways for ROS, SA, and carotenoids are especially active in the resistant cultivar. These findings could lead to a better understanding of the GLS resistance mechanisms and facilitate the breeding of GLS-resistant maize cultivars.

**Electronic supplementary material:**

The online version of this article (10.1186/s12864-018-5072-4) contains supplementary material, which is available to authorized users.

## Background

Gray leaf spot (GLS), which is caused by the necrotrophic fungi *Cercospora zeae-maydis* and *Cercospora zeina*, is one of the most serious foliar diseases in maize among almost all maize-growing regions worldwide [1, 2]*. Cercospora* spores overwinter on maize plant debris in diseased fields, and conidia begin to germinate under suitable climatic conditions (i.e., warm temperatures and high humidity) in the next planting season [3, 4]. The disease symptoms are characterized by distinct rectangular shapes parallel to the veins [5], and the severity of GLS is highly dependent upon climate conditions and cultivars, which could cause yield losses of over 70% owing to severe blight, stalk deterioration, and lodging [2, 6]. This disease has also occurred commonly in Sichuan Province, China since 2007 [7] owing to a lack of resistant cultivars and the temperate mountain climate, which favors the spread of this disease.

Compared with regular methods for disease control by chemicals, cultivation of GLS-resistant hybrids is urgently needed, as it is a cost-effective and environmentally friendly approach for reducing yield loss caused by GLS. Thus, a better understanding of the genetic architecture and the underlying genes and mechanisms could contribute to crop improvement and disease management. In general, plant immunity to necrotrophic infections is associated with complex biological and physiological processes [8], including host cell death [9, 10] and the production of various secondary metabolites [11, 12] and hormones [13–15] as well as the accumulation of reactive oxygen species (ROS) [16, 17] and a variety of cell wall modifications [14, 18]. Previous studies have been focused on GLS resistance QTL mapping and a variety of genetic loci were identified [19–21]. QTL mapping of a maize recombinant inbred line (RIL) population derived from subtropical CML444 × SC Malawi maize inbred lines identified seven GLS-resistance QTL in total from five sites, and each of two QTLs (located in bin 4.08 and bin 6.06/6.07) explained more than 11% of the phenotypic variation [22]. The maize nested association mapping (NAM) population was used to identify three QTLs that reduced GLS disease incidence by more than 10%, and experiments with near isogenic lines were conducted to develop hypotheses about resistance mechanisms [19]. Bi-parental QTL mapping was combined with GWAS to refine the map positions of GLS-resistance QTLs on chromosomes 1, 6, 7, and 8. One QTL was fine-mapped to a region with 15 candidate genes [21]. An integrated systems genetics approach recently identified four hotspots that coincide with GLS severity QTLs located on chromosomes 4, 9, and 10 [23]. Experiments with a maize RIL population in South Africa supported a hypothesized network associated with susceptibility in the maize–GLS pathosystem. However, no GLS resistance genes have been cloned, and the knowledge of maize resistance to GLS is still limited.

RNA-Seq can provide an efficient way to assess the global expression variation in coding genes and identify gene-based markers at the whole genome level. Recently, RNA-Seq analysis of resistant and susceptible sub-tropical maize lines showed that zealexins and kauralexins are accumulated after infection by *C. zeina* of both germplasms adapted to the southern African climate [24]. Several candidate genes for the gibberella ear rot resistance were identified by screening global gene expression profiles for differentially expressed genes mapped to within resistance QTL regions [25]. RNA-Seq was also used to identify SNPs that provide a useful resource for genetic and genomic studies and are a valuable asset for development of functional markers in soybean [26].

‘Yayu889,’ as a maize cultivar with high proven resistance to GLS in field conditions of China, renders itself an excellent material for understanding maize resistance to GLS. To facilitate our analysis, the susceptible cultivar ‘Zhenghong532’ was used as a reference strain. The transcriptional response of these two maize cultivars to GLS was analyzed during four stages of infection by using RNA-Seq, and both common and specific differentially expressed genes (DEGs) associated with the defense response to GLS in the two cultivars were investigated. To the best of our knowledge, this study is the first to report the induced defense response to GLS in maize by using a comparative transcriptome analysis. By screening global gene expression profiles for DEGs followed by quantitative analysis of protein and comparisons to resistance QTL regions previously reported, we have identified candidate genes for GLS resistance. Moreover, we found several pathways involved in maize responses to GLS by metabolic analysis. This may ultimately help to reveal the molecular mechanisms of maize–GLS interactions, which forms an important step towards developing molecular markers-assisted selection (MAS) for GLS-resistant genetic materials.

## Methods

### Germplasm and field trials

Two commercial maize lines, ‘Yayu889’ and ‘Zhenghong 532,’ were used in our study, and both of them were provided by Sichuan Nongda Zhenghong Bio. Co., Ltd., China. Both of them were planted in Guangyuan (N 32°36′ 19.96″, E 106°05′ 59.34″; 1354 masl), Sichuan Province, China, where GLS outbreaks occur every year. For each line, seeds were sown in three replicate rows of 10 plants. Before this study, a natural infection experiment was conducted for three consecutive years in the field, confirming that ‘Yayu889’ and ‘Zhenghong532’ were resistant and susceptible, respectively.

Leaf samples of three biological replicates were collected at 81, 89, 91, and 93 days post planting (dap) and stored in liquid nitrogen. Disease symptoms were evaluated at 81, 89, 91, 93, and 123 dap, respectively, based on a 1–5 scale with 0.25 increments, according to disease progression on the ear leaf [27].

### cDNA library construction and sequencing

Total RNA was isolated and purified from leaf tissues with TRIzol reagent (Invitrogen, Carlsbad, CA, USA) according to the manufacturer’s instructions. To ensure that the RNA meets the requirements for transcriptome sequencing, a Nanodrop spectrophotometer (Thermo Fisher Scientific Inc., Wilmington, DE, USA), Qubit RNA Kit (Life Technologies, Carlsbad, Ca, USA), and 2100 Bioanalyzer (Agilent Technologies, Santa Clara, CA, USA) were used to determine the purity, concentration, and integrity of samples, respectively.

Both cDNA library preparation and sequencing were conducted by the Biomarker Technology Company in Beijing, China. All 24 libraries were sequenced using an Illumina HiSeq™4000 (Illumina, San Diego, CA). The raw data were filtered with the FASTQ_Quality_Filter tool from the FASTX-toolkit. After preprocessing the RNA-Seq data, the reads were mapped to the maize reference genome version 4 (B73 RefGen_v4) using Tophat2 [28], which was download from ftp://ftp.ensemblgenomes.org/pub/release-32/plants/fasta/zea_mays/dna/. The sequence Alignment generated by Tophat2 was then processed by the software package Cufflinks to assemble the Sequence Alignment/Map file into transcript fragments. FPKM [29] was used as the unit of measurement to estimate transcript abundance.

### Differential expression analysis

Differential expression analysis was performed using the DESeq R package (1.10.1) [30]. DESeq provides statistical routines for determining differential expression in RNA-Seq gene expression data using a model based on a negative binomial distribution. The resulting *P*-values were adjusted using the Benjamini and Hochberg’s approach for controlling the false discovery rate (FDR). Genes with an adjusted *P*-value < 0.05 and log_2_ (fold change) > 1 (according to DESeq) were assigned as differentially expressed.

Gene Ontology (GO) enrichment analysis of the DEGs was implemented using the GOseq R packages based on the Wallenius non-central hyper-geometric distribution [31], which can control for gene length bias in DEGs. KOBAS [32] was used to test the statistical enrichment of DEGs in KEGG pathways.

### Weighted gene co-expression network analysis

Weighted Gene Co-expression Network Analysis (WGCNA) is a statistical tool for clustering transcripts that have a similar expression pattern across a group of samples [33, 34]. WGCNA was originally developed to analyze microarray data but can also be used to examine RNA-Seq data [35]. The input data for the WGCNA were the normalized values for each transcript. First, all available samples from each time point in ‘Yayu889’ samples were collected to identify modules that had different expression patterns. Next, a soft threshold was chosen to create networks with a scale-free topology using the method described by Langfelder and Horvath [33]. After the networks were built, modules of transcripts with similar expression patterns were created and eigengenes for these modules were calculated. Finally, correlations between these eigengenes and the factor of interest (at 81 dap, 89 dap, 91 dap, and 93 dap) were calculated.

### Gene validation and expression analysis

To validate RNA-seq results, qPCR was performed. RNA samples were reverse-transcribed into cDNA using the PrimeScript® RT reagent Kit (Takara Code RR047B; Takara, Shiga, Japan). Expression profiles of genes were examined in triplicate using SYBR® Premix Ex TaqTM II (Tli RNaseH Plus; Takara Code RR820A) in LightCycler 480 (Roche Applied Science, Switzerland) following the 20-μL Real Time system, including 10 of μL SYBR® Premix Ex TaqTM II (2X), 0.6 μL of forward and reverse primer (6 μM) with a final concentration of 0.3 μM, 1.0 μL of cDNA (50 ng/μL), and 7.8 of μL sterile distilled water. Two-step PCR was performed according to the manufacturer’s procedure, and the initial denaturation step was conducted at 95 °C for 30 s, followed by 40 cycles of 95 °C for 5 s and 60 °C for 30 s, and then 72 °C for 60 s. All primers are listed in Additional file 1.

### Protein precipitation and trypsin digestion

Proteins dissolved in solution were precipitated by adding samples to three sample volumes of pre-cooled acetone (320,110, Sigma). White protein precipitates were observed upon mixing. To maximize the efficiency of protein precipitation, each sample was incubated at − 20 °C overnight (approximately 12 h). Precipitated proteins were then collected by centrifugation with a benchtop centrifuge at max speed (approximately 14 k) for 15 mins. The protein pellet was washed twice with equal volumes of pre-cooled acetone. For the washing step, a shorter centrifugation time of 5 mins was used. Residual acetone was removed by drying in a biosafety cabinet or light blowing with air.

Proteins were re-suspended in 8 M urea and reduced with 20 mM DTT at 60 °C for 1 h. Proteins were then alkylated with 40 mM IAA at room temperature for 30 min in darkness. Alkylation reactions were quenched with 10 mM DTT. Samples were diluted to 2 M urea concentrations with HPLC grade water. Protein concentrations of the samples were determined with a modified Lowry’s assay (DC Assay Kit, Cat. 500–0111, Bio-Rad, Hercules, CA, USA) with the use of bovine serum albumin (BSA) to construct a calibration curve. An appropriate amount of trypsin was then added to the samples to reach an enzyme-to-substrate ratio of 1:100. Digestion was performed with 100 mM triethylammonium bicarbonate (pH 8, T7408, Sigma-Aldrich, St. Louis, MO, USA) at 37 °C for 18 h. Digested proteins were desalted with ZipTip (Cat. ZTC18S960, Millipore, Billerica, MA, USA) for LC-MS/MS analysis.

### Protein quantification using the multiple reaction monitoring (MRM) method

Separation of protein digests was performed on an Eksigent NanoLC-Ultra® 2D System and cHiPLC® system (Eksigent, Dublin, CA, USA) in serial column mode. For each injection, the sample was desalted on a 75 μm × 15 mm analytical column, then eluted onto a second analytical column to create a 30-cm column length for separation. Both column chips were filled with ChromXP™ C18-CL 3 μm 120 Å phase (Eksigent, Dublin, CA, USA). Peptides were separated using a linear gradient formed by A (2% ACN, 0.1% FA) and B (98% ACN, 0.1% FA), from 12 to 32% of B over 60 min at a flow rate of 250 nL/min. Each injection was performed using a full loop injection with a 1-μL sample loop. The MS analysis was performed on a TripleTOF® 5600 system (AB Sciex, Framingham, MA, USA) using both the MRM^HR^ workflow and Scheduled MRM^HR^ workflow. High resolution mode (> 30,000) was used to capture MS spectra with the accumulation time being 250 ms per spectra. We performed full scan MS/MS under high-sensitivity mode, and optimized the accumulation time per-cycle. Rolling collision energy was used to set collision energy that spread of 5 V. The retention time windows used for datasets were 2.5 min.

ProteinPilot (Version 4.5.0.0 by AB Sciex) was used for sample profiles with the Paragon method to source possible peptide matches from the UniProt protein database (release 2013_08). The following search parameters in the Paragon algorithm in ProteinPilot were used: Sample type, identification; Cys alkylation, Iodoacetamide; Digestion, trypsin; Instrument, TripleTOF 5600; Special Factors, none; and Search Effort, Thorough ID. FDR analysis in the ProteinPilot software was performed, and FDR < 1% was used for the protein identification threshold. The candidate peptides were verified by using PeptideAtlas (www.peptideatlas.org), and the XICs for specific m/z values of different peptides were viewed by PeakView (version 1.1.1.2 by AB Sciex). The MS/MS spectrum detected by the mass spectrometer and the theoretical fragmentation spectrum for the peptides were manually checked to confirm the presence of the peptide in the XIC profile. Multiquant (version 2.0.2 by AB Sciex) was used to calculate peak area integration for the top three transitions selected from the MS/MS spectra.

### Validation of SNP markers in candidate proteins

The SAMtools software package was used to simultaneously call SNPs across all samples [36]. The results were filtered to discard SNPs with quality scores below 70. To ensure reliability of these SNPs, those identified in at least eight replicates (out of 12 libraries) for a cultivar that had a total read depth of at least six were assigned as SNPs for that cultivar. The identified SNPs from RNA-Seq were selected for PCR amplification and Sanger sequencing. Flanking sequences of selected SNPs were extracted from the reference genome and PCR primers were designed with Premier Primer 5 (Premier Biosoft, Palo Alto, CA, USA). All primers are listed in Additional file 1.

### SA content determination assay

Leaf tissues with fresh weights of 100 mg were homogenized and extracted in 900 μL of 0.05 M phosphate saline buffer (pH, 7.4). The homogenate was centrifuged at 1 × 10^4^ rpm at 4 °C for 20 min, and the supernatant was collected. Then, the levels of salicylic acid (SA) were assessed using enzyme linked immunosorbent assay (ELISA) test kits (Hermes Criterion Biotechnology, Vancouver, Canada) according to the manufacturer’s instructions.

### Peroxidase enzyme activity assay

The crude enzyme extraction method was the same as those outlined above. Peroxidase (POD) activity was determined using the method of Upadhyaya et al. [37] with minor modification. The reaction mixture contained 3.9 mL of 0.3% guaiacol, 50 μL of enzyme extract, and 50 of μL 0.3% H_2_O_2_. The absorbance was monitored at 470 nm for at least 5 min at 1-min intervals; an absorbance change of 0.01 represented one unit of POD activity.

### Carotenoid content determination assay

The total carotenoids were determined by the method of Yang et al. [38] with slight modification. Leaf tissues with fresh weights of 30 mg were ground and extracted in 1 mL of an acetone–water mixture (4:1). Total carotenoids were also calculated using the Yang et al. method [38].

### Statistical analysis

Data obtained in the experiments were used to evaluate the disease index and MRM were subjected to an analysis of variance (ANOVA) using the Statistical Package for Social Science (SPSS; SPSS Inc., Chicago, IL, USA) version 17.0. The statistical significance was judged at a threshold of *P* < 0.05.

## Results

### Evaluation of maize cultivars against GLS

‘Yayu889’ and ‘Zhenghong532’ were cultivated in 2012 and 2011, respectively, in southwestern China. They showed similar growth rates and developmental progressions throughout the sampling period. The growing cycle from sowing to tassel lasted 98 days for ‘Yayu889’ and 100 days for ‘Zhenghong532.’ The growing cycle from sowing to maturity lasted 170 days for ‘Yayu889’ (plant height, 280 cm; ear length, 19.8 cm) and 163 days for ‘Zhenghong532’ (plant height, 240 cm; ear length, 19.5 cm), while different resistance levels were exhibited in response to GLS. The development of disease symptoms of the two maize cultivars was investigated at 81, 89, 91, and 93 dap, respectively, and GLS symptoms were scored at 123 dap.

**Fig. 1:**
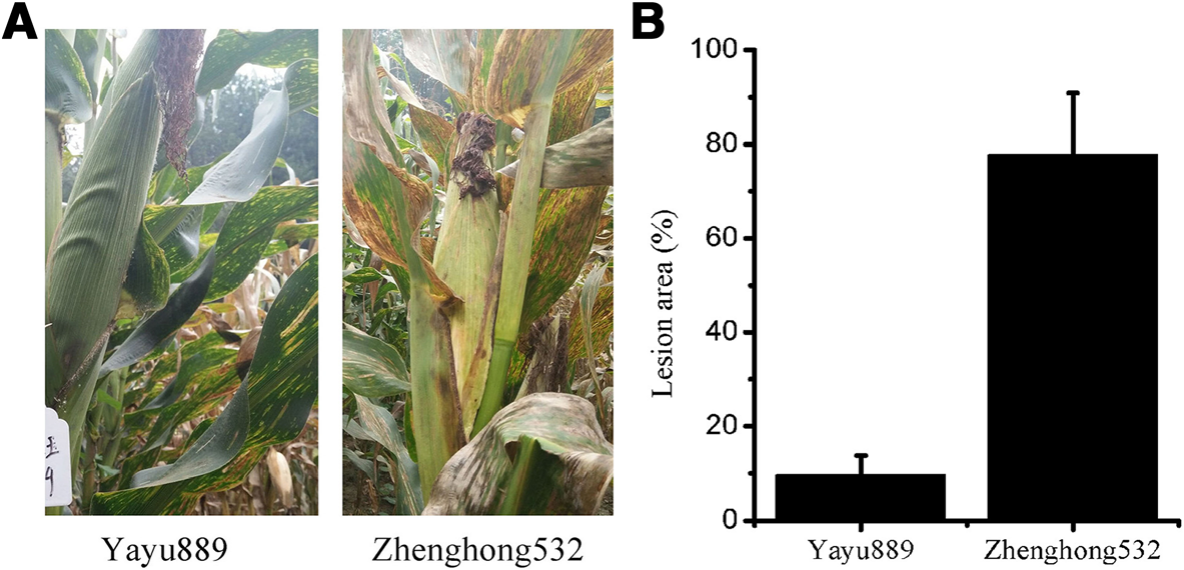
Evaluation of two maize cultivars against gray leaf spot disease (GLS). (**a**) Image of representative ‘Yayu889’ and ‘Zhenghong532’ plants from a field of cultivated maize infected with GLS. (**b**) Lesion area of GLS infections in ‘Yayu889’ and ‘Zhenghong532.’ Data are represented as means ± SEMs

Of the two cultivars evaluated against GLS, ‘Yayu889’ was resistant (disease index, 14.9%), while ‘Zhenghong532’ was susceptible (disease index, 78.4%; Fig. 1). We first observed small chlorotic spots on the bottom leaves at 89 dap. Then, spots developed into small tan spots with chlorotic borders at 91 dap. At 93 dap, mature lesions were formed, which were gray to tan in color and distinctly rectangular in shape. According to the observed phenotypic change, we sampled tissues at three time points (89, 91, and 93 dap) with typical lesions and at the beginning time point (81 dap) with no disease symptoms to investigate the differential transcript changes after challenging ‘Yayu889’ and ‘Zhenghong532’ with GLS, respectively. Three biological replicates were sequenced for each genotype and time point. The beginning time point (81 dap) was used as control group for profiling the induced transcription changes under GLS in both cultivars.

### Differentially expressed genes

A total of 628.95 million reads were generated via 150-bp paired-end sequencing from 24 cDNA libraries. The raw data were filtered with the FASTQ_Quality_Filter tool from the FASTX-toolkit (Additional file 2). The clean data were used for further analysis. After preprocessing the RNA-Seq data, the reads were mapped to the maize reference genome version 4 (B73 RefGen_v4) using Tophat2 [28] (Additional file 3). Differential expression analysis was performed using the DESeq R package (1.10.1).

By comparing the RNA-Seq expression profiles of each pair of three biological replicates (Additional file 4), the correlation coefficients were evaluated. The results showed that three biological replicates in each group had high consistency.

**Fig. 2:**
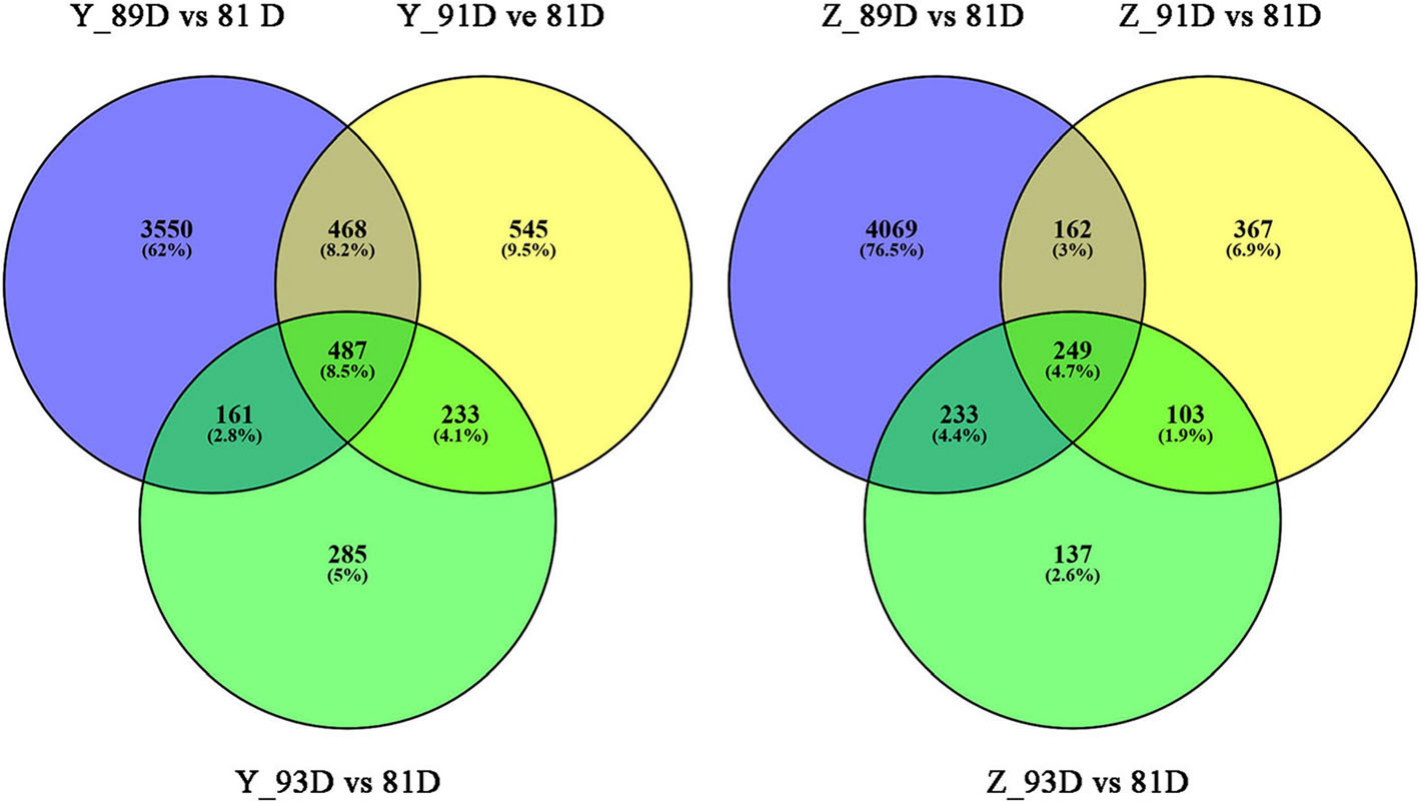
Venn diagrams of differentially expressed genes (DEGs) modulated by gray leaf spot disease (GLS). Venn diagrams represent DEGs in ‘Yayu889’ and ‘Zhenghong532’ challenged by GLS at 89, 91, and 93 days post planting. Y and Z represent ‘Yayu889’ and ‘Zhenghong532,’ respectively; D represents days post planting

The number of DEGs at the three disease phases in ‘Yayu889’ were 4666, 1733, and 1166, respectively (Yayu889 _ 81 dap vs Yayu889 _ 89 dap, Yayu889 _ 81 dap vs Yayu889 _ 91 dap, and Yayu889 _ 81 dap vs Yayu889 _ 93dap), while 487 genes were common DEGs among all three time points in ‘Yayu889’ (Fig. 2). The number of DEGs at each of the three disease phases in Zhenghong532 were 4713, 881, and 722, respectively (Zhenghong532 _ 81 dap vs Zhenghong532 _ 89 dap, Zhenghong532 _ 81 dap vs Zhenghong532 _ 91 dap, Zhenghong532 _ 81 dap vs Zhenghong532 _ 93dap), while 249 genes were common DEGs among all three time points in ‘Zhenghong532’ (Fig. 2).

Functional annotations of the DEGs available from seven public databases were used to investigate the functions and roles in response to GLS. Several categories of defense-related genes, which were already reported in plant immunity to necrotrophic fungi infection [39–44], are described in the following sections.

**Fig. 3:**
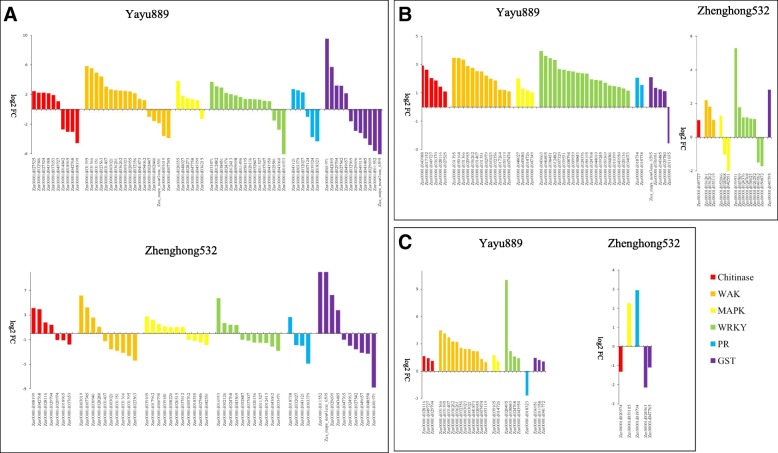
Transcriptome profiling of defense-related genes from ‘Yayu889’ and ‘Zhenghong532’ in response to gray leaf spot disease (GLS) infections. Three infected stages of two maize cultivars are shown: (**a**) 89 days post planting, (**b**) 91 days post planting, and (**c**) 93 days post planting

At 89, 91, and 93 dap we found ten, six, and three chitinases that were differentially expressed in ‘Yayu889,’ while seven, one, and one were up-regulated in ‘Zhenghong532,’ respectively (Fig. 3, Additional file 5). One up-regulated chitinase was common in ‘Yayu889’ at all three time points. Interestingly, one chitinase (Zm00001d037653) was up-regulated in ‘Yayu889’ at 89 dap, but down-regulated in ‘Zhenghong532’ at the same phase. In ‘Yayu889,’ we found six, two, and one differentially expressed PR genes at 89, 91, and 93 dap, respectively, while 4, 0, and 1 PR genes were differentially expressed in ‘Zhenghong532,’ respectively, and three up-regulated PR genes (Zm00001d003379, Zm00001d032027, and Zm00001d043121) in ‘Yayu889’ were down-regulated in ‘Zhenghong532’ at 89 dap (Fig. 3, Additional file 6). ‘Yayu889’ exhibited 19, 14, and 12 wall associated kinases (*WAK*) at 89, 91, and 93 dap, respectively, and seven of them were shared among all three assayed phases (Fig. 3, Additional file 7). ‘Zhenghong532’ exhibited only ten, three, and zero *WAK*, respectively. Notably, out of seven common up-regulated *WAK* genes in ‘Yayu889,’ three (zm00001d031366, Zm00001d031395, and Zm00001d031407) were down-regulated in ‘Zhengong532’ at 89 dap and exhibited baseline expression at 91 and 93 dap. Six, four, and two mitogen-activated protein kinases (*MAPK*) were identified in ‘Yayu889’ at 89, 91, and 93 dap, respectively, while ten, three, and one *MAPK* genes were found in ‘Zhenghong532’ (Fig. 3, Additional file 8). Interestingly, out of the five up-regulated *MAPK* genes in ‘Yayu889’ at 89 dap, two (Zm00001d020355 and Zm00001d048027) were down-regulated in ‘Zhenghong532’ at the same phase. ‘Yayu889’ exhibited induction of 16, 20, and 4 *WRKY* genes at 89, 91, and 93 dap, respectively. In contrast, ‘Zhenghong532’ exhibited induction of only eleven, eight, and zero WRKY genes, respectively (Fig. 3, Additional file 9). We found, respectively, five, four, and three up-regulated glutathione *S*-transferase (*GST*) genes in ‘Yayu889’ at 89, 91, and 93 dap. While the number of up-regulated *GST* genes was four, one, and zero in ‘Zhenghong532’ (Fig. 3, Additional file 10). Interestingly, out of the five up-regulated *GST* genes in ‘Yayu889’ at 89 dap, three (Zm00001d027542, Zm00001d049657, and Zm00001d001971) were down-regulated in Zhenghong532 at 89 dap.

### Quantitative RT-PCR validation

**Fig. 4:**
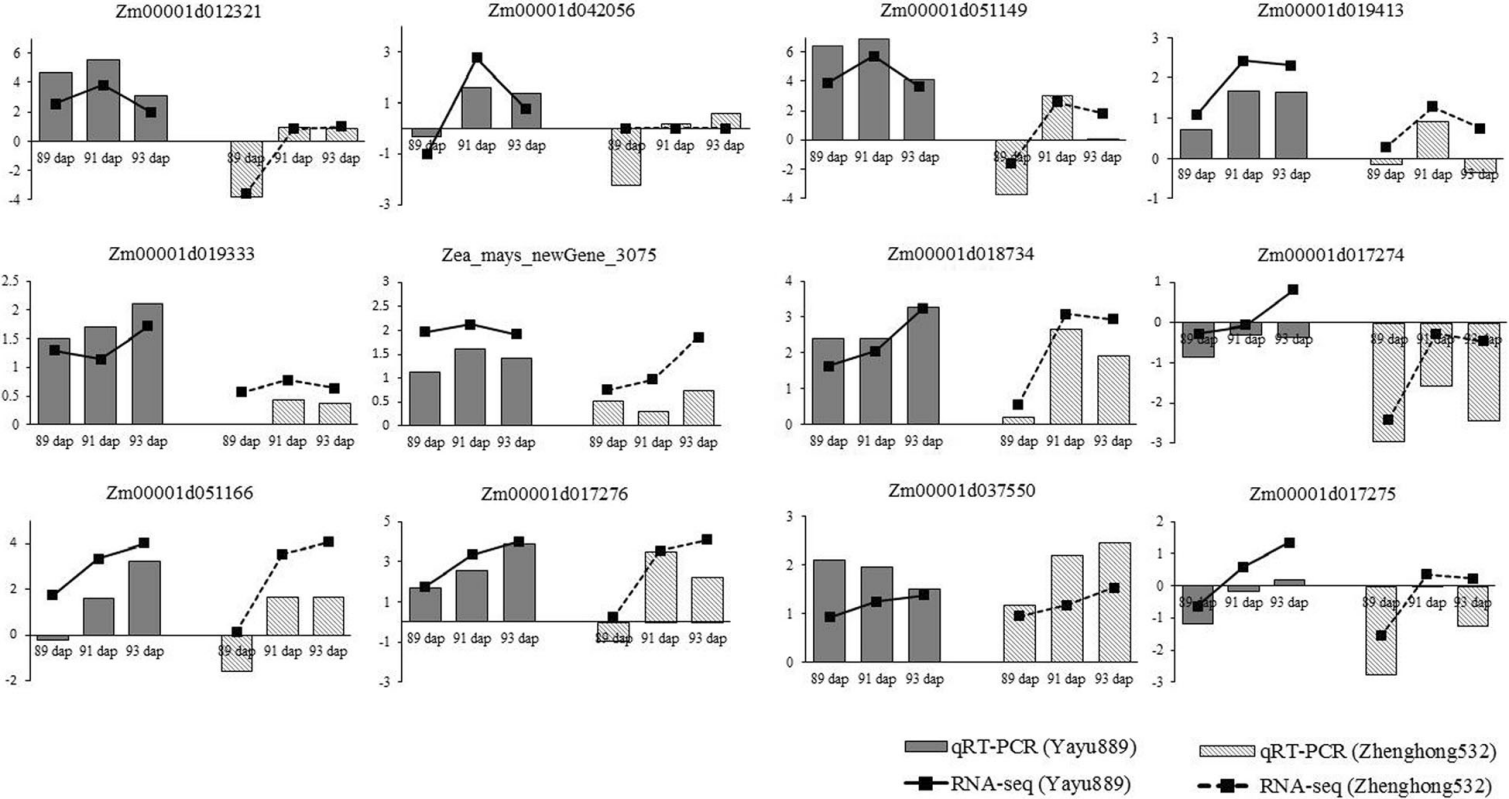
Validation of RNA-seq data by qRT-PCR. Twelve differentially expressed genes (DEGs) were selected for validation, and they showed a similar tendency with RNA-Seq. The *y*-axis showed the fold changes of three disease stages compared to the beginning point, with positive values indicating up-regulation and negative values indicating down-regulation. Each data point was obtained from three biological replicates

To validate the RNA-Seq data, 12 DEGs were randomly selected for qRT-PCR assays based on their expression patterns at three time points. The results of the selected DEGs showed that the qRT-PCR results were consistent with the RNA-Seq results, as both RNA-Seq and qRT-PCR analyses showed similar expression patterns of up- and down-regulation, indicating that the RNA-seq analysis was well suited for analysis of GLS-induced maize leaf transcriptomes (Fig. 4, Additional file 1).

### GO and KEGG enrichment analyses

**Fig. 5:**
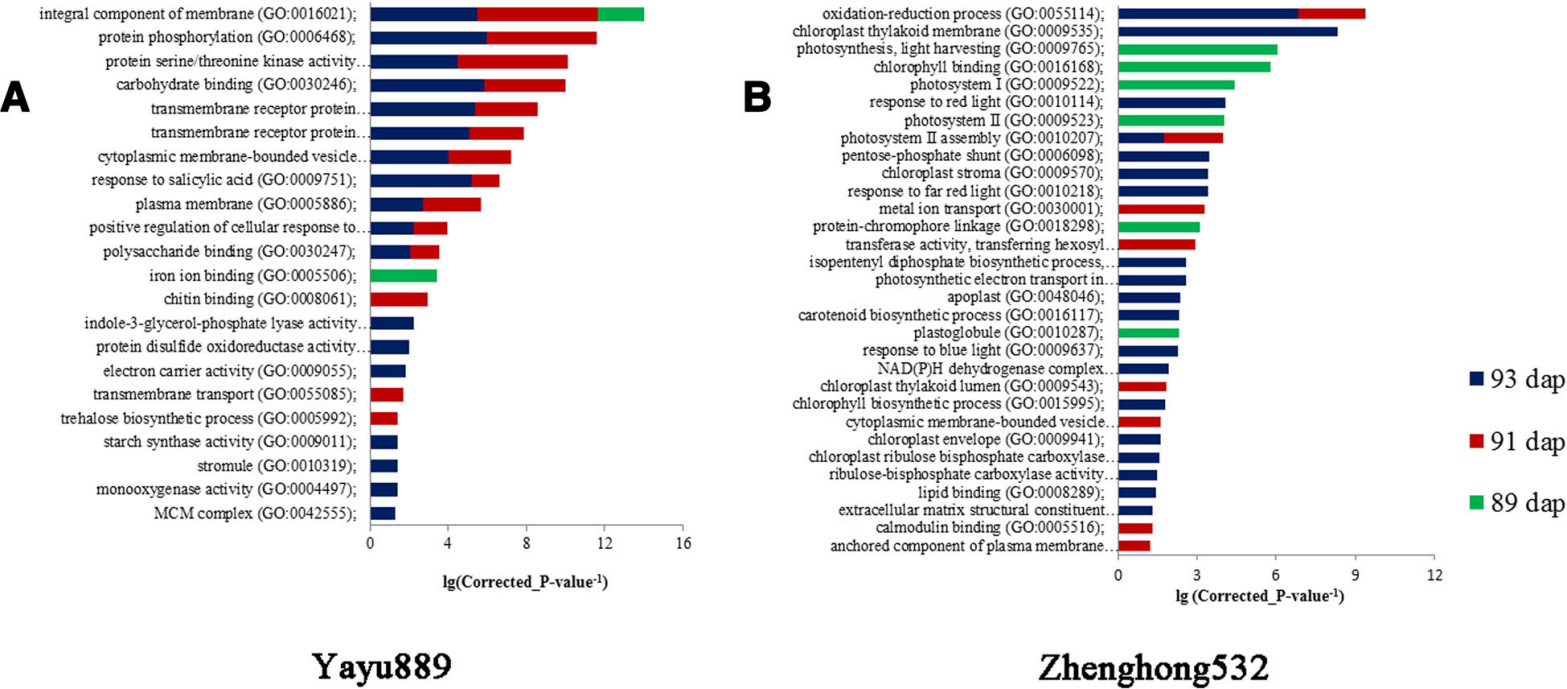
GO enrichment analyses in ‘Yayu889’ and ‘Zhenghong532’ after gray leaf spot disease (GLS) infection. Common and genotype-specific enriched GO terms were obtained with the goseq package using a false discovery rate cutoff of 0.1. GO term enrichment *P*-values are indicated on the *x*-axis. Blue, red, and green columns refer to biological process (BP), molecular function (MF), and cellular component (CC) GO domains, respectively

GO category enrichment analysis was applied to elucidate the functional enrichment of the DEGs and Fisher’s exact test when a corrected *P*-value < 0.05 was used. A total of 22 GO terms were enriched in the resistant ‘Yayu889’ cultivar, of which 11 common GO terms were significantly enriched at 91 and 93 dap (Fig. 5a). Several of the 22 common GO terms in ‘Yayu889’ were associated with general plant responses to fungal attacks, including ‘response to salicylic acid’ (GO:0009751), ‘protein phosphorylation’ (GO:0006468), ‘transmembrane receptor protein serine/threonine kinase signaling pathway’ (GO:0007178), and ‘chitin binding’ (GO:0008061). In the susceptible ‘Zhengong532’ cultivar, there were 31 significantly enriched GO terms, while only two common GO terms were enriched at 91 and 93 dap (Fig. 5b). Although ‘Zhenghong532’ displayed a higher number of GO terms, many of them were not associated with a specific plant response to biotic stress. Furthermore, a different battery of response mechanisms to GLS was activated by ‘Zhenghong532’ compared to ‘Yayu889,’ e.g., ‘oxidation-reduction process’ (GO:0055114) and ‘carotenoid biosynthetic process’ (GO:0016117).

In order to compare and summarize the response of the two cultivars to GLS, the DEGs were mapped to the reference canonical pathways in KEGG. Based on the plant–pathogen interaction map, most DEGs were involved in the hypersensitive response and Ca^2+^ signaling pathways. ‘Yayu889’ expressed more Ca^2+^-dependent protein kinase (*CDPK*) genes and *CML* genes from these two pathways compared to ‘Zhenghong532’ (Additional file 11). As summarized in the plant–pathogen interaction pathway, four *CML* genes (Zm00001d005766, Zm00001d042056, Zm00001d029028, and Zm00001d003197) were specifically up-regulated in ‘Yayu889’ (91 dap), while baseline expression was exhibited in ‘Zhenghong532’ (Additional file 11), which emphasizes the possible role of Ca^2+^-dependent signaling in the resistance of ‘Yayu889’ to GLS.

### Weighted gene co-expression network analysis (WGCNA)

**Fig. 6:**
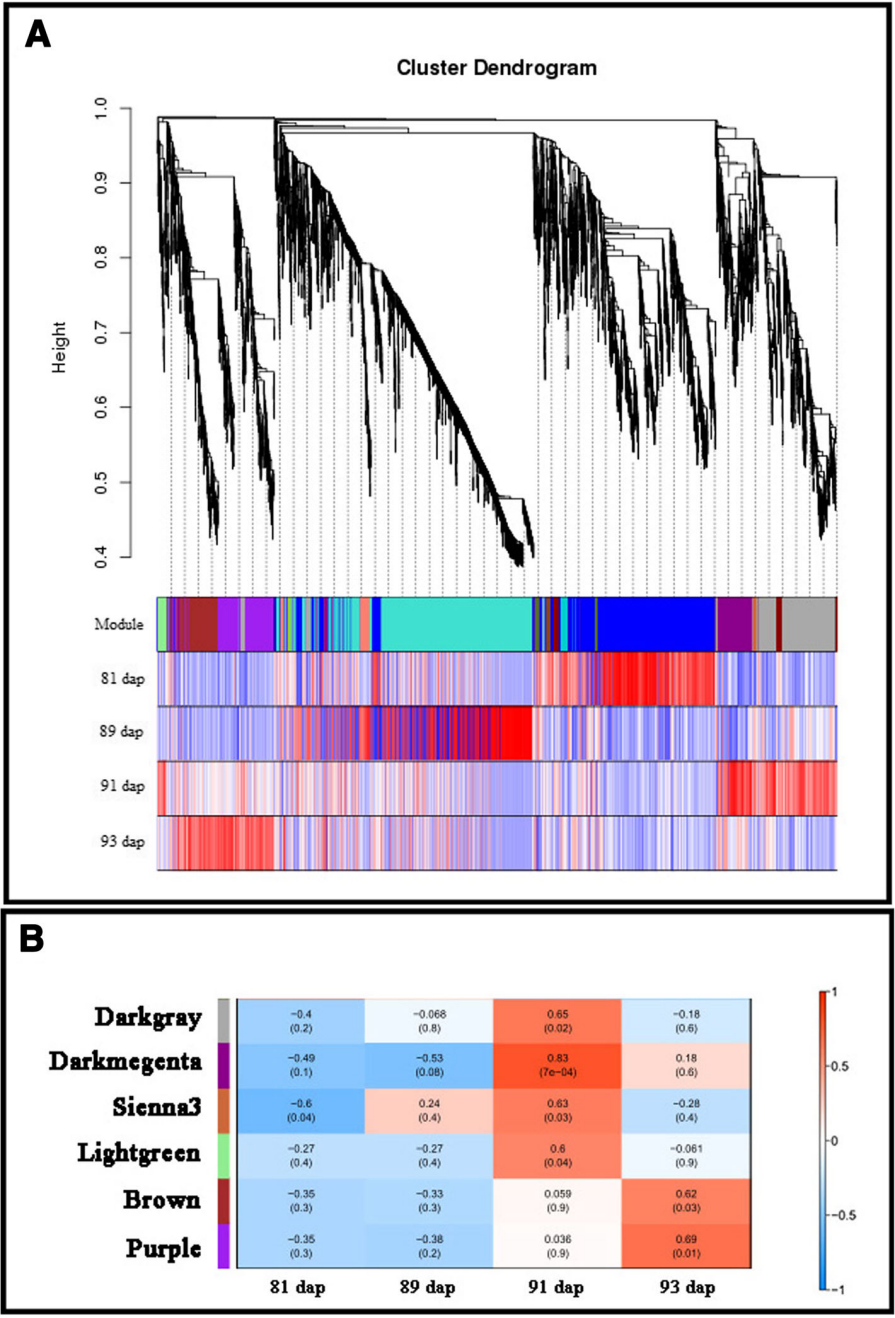
Weighted Gene Co-expression Network Analysis (WGCNA) of the transcripts changes in ‘Yayu889.’ (**a**) Hierarchical cluster dendrogram using transcripts identified by WGCNA in ‘Yayu889’ at different infection stages across 13 co-expression modules. (**b**) Module trait correlation analysis showed that six modules were significantly correlated with GLS disease expression

To determine whether coordinated transcriptional responses to GLS were correlated with resistance in ‘Yayu889,’ the datasets of ‘Yayu889’ expression across individual days (81, 89, 91, and 93 dap) were subjected to WGCNA. A total of 5046 transcripts were assigned to 13 co-expression modules, named after randomly assigned colors (Fig. 6a). Six of them were significantly relevant to GLS disease responses (Fig. 6b) as the eigengene was associated with the development of necrotic lesions.

**Fig. 7:**
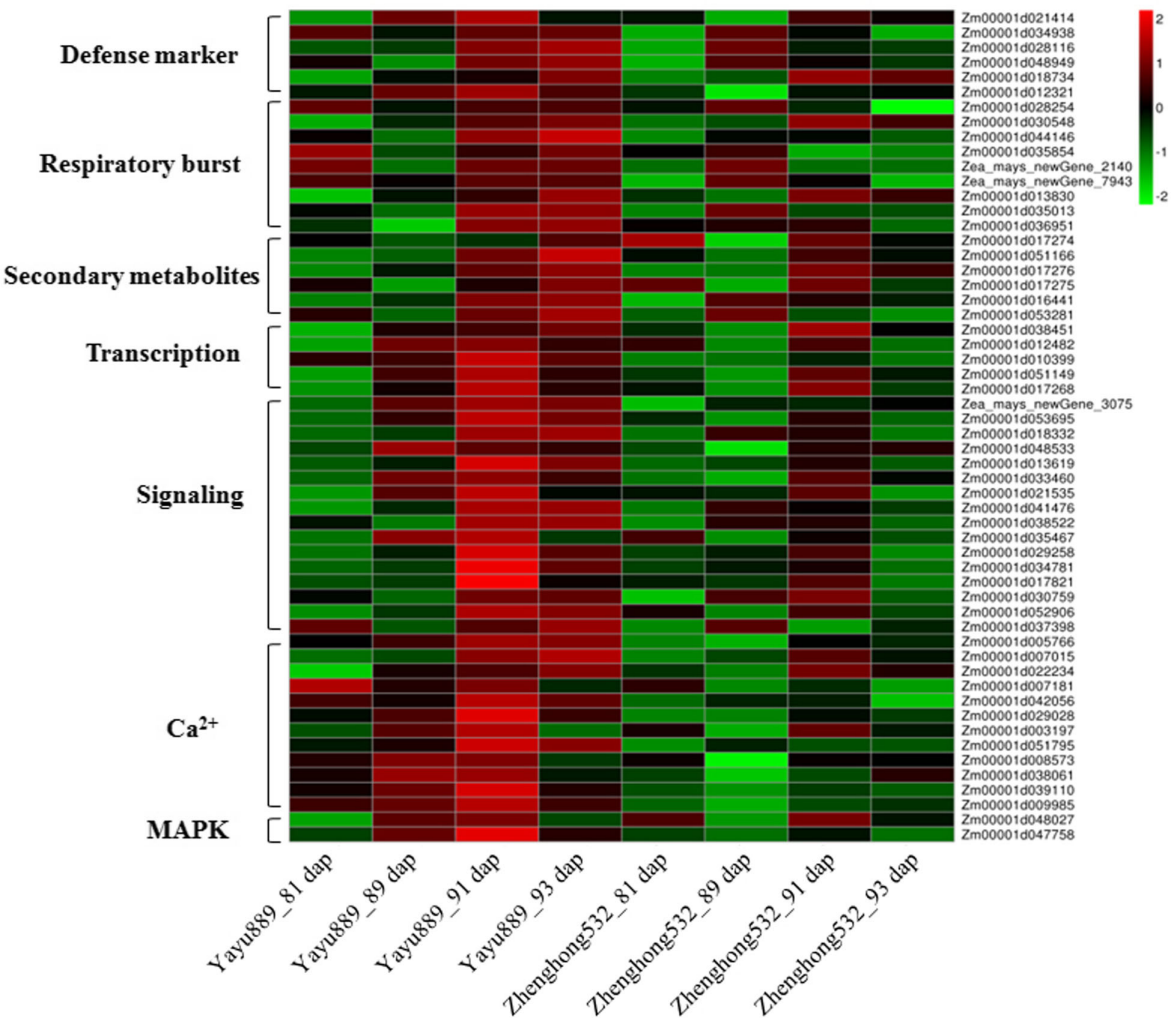
Heat maps of differentially expressed genes for putative candidate genes assigned to gray leaf spot disease (GLS)-resistance in Maize. Y and Z indicate for ‘Yayu889’ and ‘Zhenghong532,’ respectively; dap indicates day post planting; green indicates down-regulated DEGs, while red indicates up-regulated DEGs

Here, three modules (DG, DM, and PU) were listed for further analysis. We applied GO enrichment analysis to investigate the functional enrichment of transcripts in these three modules, respectively. In module DG, several biological processes related to GLS disease were enriched, including ‘protein phosphorylation,’ ‘response to chitin,’ ‘hormone-mediated signaling pathway,’ and ‘ionotropic glutamate receptor signaling’ (Additional file 12). Several genes belonging to the DG module, including two *MAPK* (Zm00001d048027 and Zm00001d047758) and *LYK5* (Zm00001d053695), were up-regulated in ‘Yayu889’ and expressed at the baseline level in ‘Zhenghong532’ (Fig. 7, Additional file 13). In the DM module, both ‘protein phosphorylation’ and ‘cellular response to hormone stimulus’ GO terms were overrepresented (Additional file 12). Two leucine-rich repeat receptor-like protein kinase genes (*LRR-RLK*; Zm00001d041476 and Zm00001d038522) of this module were significantly up-regulated at 91 and 93 dap compared to 81 dap, while baseline expression of these genes was observed at these two stages in ‘Zhenghong532’ (Fig. 7, Additional file 13). The PU module was significantly enriched for ‘vitamin E biosynthetic process,’ ‘hormone biosynthetic process,’ ‘protein phosphorylation,’ and ‘l-phenylalanine catabolic process’ (Additional file 12).

Next, multiple pathways involved in plant immunity, including ‘response to salicylic acid,’ ‘protein phosphorylation,’ ‘response to chitin,’ ‘oxidation-reduction process,’ and ‘carotenoid biosynthetic process,’ which were common or specifically enriched by differentially expressed analysis and WGCNA, were more relevant to the response to GLS in maize and thus summarized for further analysis.

### MRM quantitation of candidate proteins

GLS resistance pathways were identified by transcriptome profiling and data from the published literature, revealing a total of 56 DEGs that were associated with disease resistance (Fig. 7, Additional file 13). To develop our analysis further, we used MRM to quantify the expression of candidate proteins. As shown in Table 1, a total of 12 proteins involved in ‘response to salicylic acid,’ ‘protein phosphorylation,’ and ‘response to chitin’ were quantified. In ‘Yayu889,’ two of these proteins (CML25 and CML42) were significantly up-regulated at 93 dap compared to 81 dap, while baseline expression was viewed at all three stages in ‘Zhenghong532,’ confirming the role of CML in GLS responses. The remaining 10 proteins showed a trend of up-regulation in both genotypes, but the expression level of ‘Yayu889’ was significantly higher than that in ‘Zhenghong532’ at all four time points, including two CML proteins, two LRR-RLK proteins, RING-H2 finger protein ATL2A, nematode resistance protein-like HSPRO2, putative serine/threonine-protein kinase-like protein CCR3, protein LYK5, MAP kinase kinase (MKK), and glutathione-*S*-transferase (GST).

**Table 1:** Quantitation of candidate genes by multiple reaction monitoring (MRM)

ID	Protein description	Nomalized Expression							
		Z_81 dap	Z_89 dap	Z_91 dap	Z_93 dap	Y_81 dap	Y_89 dap	Y_91 dap	Y_93 dap
Zm00001d005766	CML	1.55 ± 0.29^a^	2.68 ± 0.35^b^	4.45 ± 0.11^c^	4.41 ± 0.1^c^	5.11 ± 0.33^cd^	5.64 ± 0.39^d^	5.82 ± 0.08^d^	6.71 ± 0.28^e^
Zm00001d042056	CML38	1.60 ± 0.32^a^	2.57 ± 0.43^a^	6.85 ± 0.34^b^	6.7 ± 0.22^b^	7.84 ± 0.48^bc^	9.34 ± 1.98^bd^	9.74 ± 0.34^cd^	15.63 ± 0.9^e^
Zm00001d038061	CML25	1.26 ± 0.13^a^	1.7 ± 0.23^a^	3.28 ± 0.09^ab^	2.90 ± 0.1^ab^	4.74 ± 0.17^bc^	5.14 ± 1.62^bc^	5.43 ± 0.08^c^	7.93 ± 0.73^d^
Zm00001d029028	CML42	1.34 ± 0.18^a^	2.94 ± 0.26^ab^	2.85 ± 0.07^ab^	4.14 ± 0.15^ab^	6.09 ± 0.5^bc^	9.37 ± 4.04^cd^	9.65 ± 0.92^cd^	11.31 ± 0.81^d^
Zm00001d021414	ATL2A	1.65 ± 0.34^a^	2.82 ± 0.43^a^	5.42 ± 0.25^b^	5.33 ± 0.19^b^	6.33 ± 0.14^bc^	7.91 ± 1.23^cd^	11.07 ± 0.36^e^	9.19 ± 0.66^d^
Zm00001d012321	HSPRO2	1.69 ± 0.36^a^	3.23 ± 0.41^a^	6.45 ± 0.24^b^	6.50 ± 0.24^b^	8.4 ± 0.32^bc^	10.51 ± 2.03^cd^	12.51 ± 0.47^de^	13.82 ± 0.68^e^
Zm00001d036951	GST	1.70 ± 0.37^a^	2.76 ± 0.5^a^	6.48 ± 0.31^b^	6.30 ± 0.24^b^	7.93 ± 0.4^bc^	10.05 ± 2.22^cd^	10.66 ± 0.23^ce^	12.28 ± 1^de^
Zm00001d038522	LRR-RLK	1.87 ± 0.46^a^	3.76 ± 0.47^a^	5.24 ± 0.29^ab^	7.35 ± 0.19^bc^	10.04 ± 0.24^cd^	12.95 ± 2.34^de^	17.25 ± 0.48^f^	15.4 ± 2.06^ef^
Zm00001d041476	LRR-RLK	1.78 ± 0.41^a^	2.54 ± 0.42^a^	6.01 ± 0.37^b^	6.63 ± 0.28^b^	7.69 ± 0.19^b^	10.64 ± 1.72^c^	13.61 ± 0.34^d^	12.16 ± 1.19^cd^
Zm00001d035467	CCR3	2.02 ± 0.55^a^	4.03 ± 0.8^ab^	8.6 ± 0.55^bc^	11.7 ± 0.51^cd^	14.12 ± 0.22^d^	18.92 ± 2.86^e^	22.56 ± 0.41^ef^	26.36 ± 3.19^f^
Zm00001d053695	LYK5	1.60 ± 0.31^a^	2.66 ± 0.44^a^	5.8 ± 0.17^b^	5.15 ± 0.16^b^	5.34 ± 0.54^b^	7.36 ± 0.77^c^	5.93 ± 0.15^b^	7.45 ± 0.47^c^
Zm00001d048027	MKK7	1.66 ± 0.34^a^	2.86 ± 0.45^a^	6.16 ± 0.26^b^	6.35 ± 0.26^b^	8.09 ± 0.38^bc^	10.41 ± 2.11^cd^	14.3 ± 0.34^e^	12.91 ± 1.24^de^

Y and Z, ‘Yayu889’ and ‘Zhenghong532,’ respectively; dap, days post planting;

Expressions with the same lower-case letter are not significantly different (*P* > 0.05)

### Candidate gene identification

Co-localization of QTLs with DEGs facilitates the screening of candidate genes for GLS resistance, and the impact of intraspecific allelic variation on gene expression may lead to phenotypic variation, including the possibility of hybrid vigor as a beneficial trait to be exploited in crop breeding. As shown in Table 2, among the 12 candidate proteins, four (Zm00001d038522, Zm00001d038061, Zm00001d036951, and Zm00001d035467) were mapped within the GLS QTLs that were previously reported [19, 20, 22, 23]. Interestingly, all four proteins were located on chromosome 6. Then, we screened SNPs in these proteins by using the RNA-Seq data and validated them by PCR assay. As shown in Table 2, four SNPs were identified within two proteins, a non-synonymous SNP (SNP4-A) located in the exons of CML25 (Zm00001d038061) and unique to ‘Yayu889,’ while the LRR-RLK (Zm00001d038522) protein carries two unique non-synonymous SNPs (SNP1-G, SNP2-A) and a unique synonymous (SNP3-C) in ‘Zhenghong532.’ Considering that they were co-localized with resistance QTLs, these two proteins are highly likely resistance proteins for GLS disease. Moreover, another two proteins that mapped to a resistance QTL region, i.e., GST (Zm00001d036951) and CCR3 (Zm00001d035467), are also noteworthy.

**Table 2:** A list of candidate proteins with SNP markers or co-localized with QTL for GLS

Protein ID	Chr	Position (bp)	Co-localized QTL	SNP ID	SNP position (bp)	Ref	Variant	
							‘Yayu889’	‘Zhenghong532’
Zm00001d038522	6	159,140,144-159,146,200	qGLS6.06 [22, 23]	1	159,142,231	A	A	G
Zm00001d038522	6			2	159,141,338	G	G	A
Zm00001d038522	6			3	159,140,445	G	G	C
Zm00001d038061	6	146,627,006-146,627,959	qGLS6.06 [19, 22, 23]	4	146,627,690	C	A	C
Zm00001d036951	6	107,194,718-107,195,794	qGLS6.05 [19, 20]					
Zm00001d035467	6	28,070,532-28,073,409	qGLS6.01 [20]					

### Salicylic acid, peroxidases, and carotenoids are induced by GLS

**Fig. 8:**
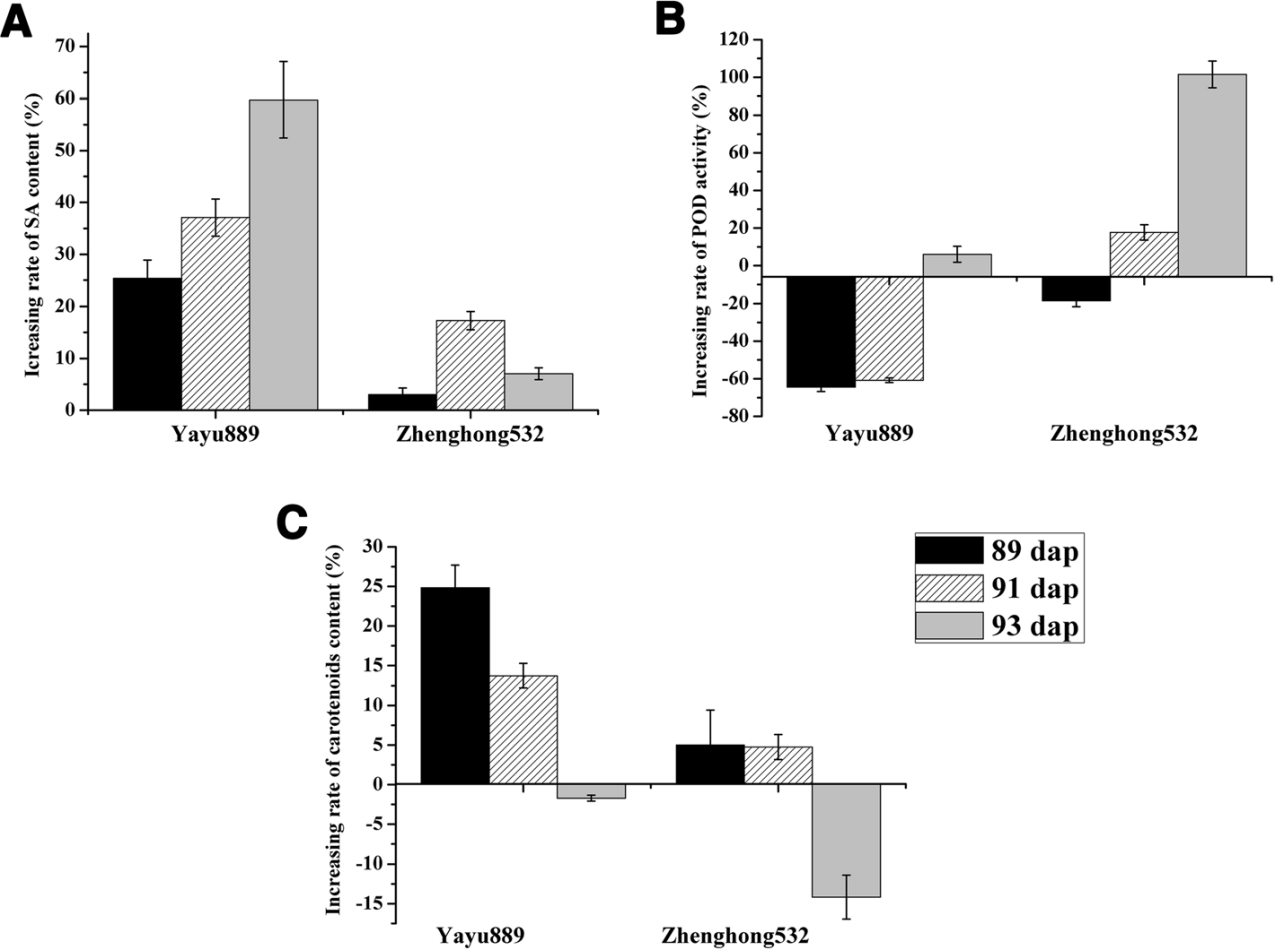
Metabolite assays. Salicylic acid (**a**), peroxidase activity (**b**), and carotenoid **(C)** assay results of maize cultivars ‘Yayu889’ and ‘Zhenghong532’ during different infection stages. Each data point was obtained from three biological replicates. Data are represented as means ± SEMs

In our study, the GO term ‘response to salicylic acid’ (GO:0009751) was specifically enriched in ‘Yayu889’ at 91 and 93 dap, and several DEGs, including two *MYB* genes, alpha-dioxygenase 1, and a *HSPRO2* gene, were identified; ‘Yayu889’ expressed a putative *MYB* gene (Zm00001d051149) at all three phases and another *MYB* gene (Zm00001d017268) at 91 and 93 dap. However, in ‘Zhenghong532,’ both of the genes only showed a weaker up-regulation at 91 dap (Fig. 4, Fig. 7, Additional file 13). Additionally, two *HSPRO2* genes (Zm00001d012321 and Zm00001d042811) that belong to this GO term were up-regulated in ‘Yayu889,’ but either down-regulated or expressed at baseline levels in ‘Zhenghong532’ (Fig. 4, Fig. 7, Additional file 13). Next, the SA content of the two maize genotypes was measured using ELISA. As shown in Fig. 8a, the SA content of ‘Yayu889’ was significantly increased at 91 and 93 dap compared to 81 dap, while no significant changes were observed in ‘Zhenghong532.’

The GO term ‘oxidation-reduction process’ (GO:0055114) was specifically found in ‘Zhenghong532.’ Protein SRG1 and four important redox enzymes of cytochrome P450, acyl-desaturase 2, geraniol 8-hydroxylase, and indolin-2-one monooxygenase were enriched. In order to verify this GO term from the metabolic level, the POD activity associated with this pathway was measured. As shown in Fig. 8b, the POD activity showed a trend of decreasing first and then increasing in both of the cultivars, but ‘Yayu889’ exhibited a greater decrease at the early infection stage compared to that of ‘Zhenghong532.’

The GO term ‘carotenoid biosynthetic process’ (GO:0016117) was overrepresented solely in ‘Zhenghong532.’ The total carotenoid content of the two cultivars was thus measured. As shown in Fig. 8c, the carotenoid level was significantly increased during early infection stages and had stabilized at 93 dap in ‘Yayu889,’ while there were no significant changes in early infection stages but a decrease by 93 dap in ‘Zhenghong532.’

These results indicate that SA, carotenoids, and POD may participate in the regulation of maize’s defense response to GLS.

## Discussion

GLS is a significant threat to maize cultivation in China owing to the current lack of resistant cultivars and the suitability of the climate of many regions to GLS. In this study, sets of genes were identified that respond to fungal infection in susceptible and resistant maize cultivars and define how each cultivar perceived the intruder and programmed its defense system. A limitation of this study is the genetic diversity between both maize lines. To reduce the impact of diverse genetic backgrounds on the results of the analysis, the datasets were filtered step by step. First, six major gene families, reported to be related to plant immunity [39–44], were found to be greatly expressed in the resistant genotype at all infection stages compared to the susceptible genotype (Fig. 3, Additional file 5). This indicates that the screened DEGs were mainly related to the disease resistance of maize. Then, bioinformatics analysis and public databases were utilized to enrich the metabolic pathways related to the plant-pathogen interactions and identify DEGs that belong to these pathways and that were potentially associated with GLS. At the same time, we further corroborated our results through metabolic and protein quantification experiments. Finally, to further narrow the screening range of resistance genes, candidate DEGs were co-localized with the resistance QTL loci for previously reported GLS [25].

Three expression modules associated with GLS responses were observed by WGCNA. The RING-H2 finger protein ATL2A, which belongs to module DG (Table 1, Additional file 12), was an early pathogen-associated molecular pattern (PAMP)-responsive gene and was reported to participate in the E3 ubiquitination degradation pathway in *Arabidopsis* [45, 46]. Another protein (LYK5) in module DG, (Table 1, Additional file 12), was reported to be a chitin receptor that could induce plant innate immunity in *Arabidopsis* [47, 48] by regulating the production of ROS and MAPK phosphorylation by interacting with the E3 ubiquitin ligase PUB13 [49]. We hypothesized that in ‘Yayu889,’ the PAMP-triggered immunity (PTI) system was activated after being challenged by GLS infection; once the pathogen invaded, LYK5 and ATL2A were activated followed by an induction of the E3 ubiquitination degradation pathway. In addition, the l-phenylalanine catabolic process in module PU, which contains four phenylalanine ammonia-lyase (PAL) proteins, was reported to participate in the biosynthesis of defense-related phytohormone SA and phytoalexin in rice [15]. Additionally, a previous study found that SA could induce the resistance of maize to GLS by enhancing PAL activity [50].

SA plays an important role in resistance and defense induction in response to pathogen attack [51]. In this study, two MYB genes (Zm00001d051149 and Zm00001d017268) were identified (Fig. 7, Additional file 13), which were reported to regulate the SA-dependent signaling pathway [52] and modulate antagonistic interactions by activating SA-mediated defenses and repressing JA-mediated defenses [53]. Additionally, *HSPRO2* (Zm00001d012321) was specifically activated in ‘Yayu889’ (Fig. 7), which was consistent with a report that *HSPRO2* positively induced the basal resistance in *Arabidopsis* against *Pseudomonas syringae* through the downstream function of SA [34]. A resistance induction test for maize exposed to GLS was previously reported, and a 30.89% induced resistance was produced by spraying SA on the leaves of maize plants [50]. Similar results were produced in our study, indicating that only the resistant cultivar ‘Yayu889’ exhibited induced SA expression (Fig. 8a). In summary, these results indicate that through modulation of the production of SA, ‘Yayu889’ regulated the level of tolerance to GLS disease, possibly through interactions of the SA signaling molecules with ROS. This was also reported under other stress conditions in other plants [54] and it was confirmed in our work by induction of POD activity in ‘Yayu889.’

A change in cellular reduction–oxidation status is one of the earliest responses detected in challenged cells [55]; Several redox enzymes were involved in this pathway. For example, maize cytochrome P450 was reported to produce phenylacetaldoxime and indole-3-acetaldoxime in heterologous systems and might contribute to plant defense [56]. Similarly, acyl-[acyl-carrier-protein] desaturase may enhance the resistance of *Arabidopsis* and soybean to multiple pathogens [57]. Additionally, GST belongs to the GSH redox system that suppresses cercosporin [58]. Lastly, production of reactive oxygen species (ROS) occurs rapidly in response to an attempted pathogen invasion of potential host plants [59]. Based on the analysis of POD activity in the present study (Fig. 8b), we hypothesized that ‘Yayu889’ induced ROS levels by inhibiting POD activity at early stages of GLS infection, such as H_2_O_2_ production and then, the inhibition of H_2_O_2_ levels by enhancing POD activity after the infection was established. This hypothesis corroborated the concept that ROS plays a role in resistance during early infection while promoting disease during the later stages of infection [8, 60].

Protein phosphorylation has been regarded as essential to plant immunity [61]. Genes within this group include various protein kinases, such as receptor kinases and *MAPK*s. In this study, two serine/threonine protein kinases were identified, which were reported to participate in a phytohormone-activated signaling pathway, and the regulation of plant-type hypersensitive response in *Arabidopsis* [62]. ‘Yayu889’ is known to encode two *LRR*-*RLK* genes (Zm00001d041476 and Zm00001d038522; Additional file 13) and two surface-localized pattern recognition receptors (PRRs). These genes were reported to constitute an important layer of innate immunity in plants. One of the genes (Zm00001d038522) contained a SNP within a major QTL region (Table 2); thus, it may be a candidate resistance gene with high potential to be applied in MAS to produce GLS-resistant genetic materials [63]. Additionally, a MKK7 protein (Zm00001d048027) in this pathway was consistently expressed at higher levels (Table 1, Additional file 13) in ‘Yayu889,’ and previous studies have found that ectopic expression of *MKK7* could elevate SA levels and enhance basal resistance to *Arabidopsis* pathogens [64].

Ca^2+^ ions are recognized as a crucial second messenger in signaling pathways, combining the perception of environmental stimuli with plant adaptive responses [65]. This conversion into biological responses requires the presence of calcium sensor relays such as calmodulin (CaM) and CML proteins [66, 67]. *CML9* and *CML8* were found to act as positive regulators of defense mechanisms against microbial pathogens in *Arabidopsis* [68, 69]. In the present study, protein expression of CML25 and CML42 was only induced in ‘Yayu889’ (Table 1). Notably, CML25 was co-localized with a major QTL region and the SNP4-A may be the reason for the difference in protein expression among the two maize cultivars (Table 2). This indicates that the Ca^2+^-dependent signaling pathway may participate in ‘Yayu889’ resistance to GLS by activation of SA and ROS, and this was verified in plant immunity to other pathogens [68, 70, 71].

Cercosporin, the non-host-selective toxin produced by *Cercospora zeina*, is a photo-activated perylenequinone that converts molecular oxygen to active oxygen species, including singlet oxygen [72]. Plant carotenoids play a key role in quenching singlet oxygen molecules and are hypothesized to play a role in defense against toxins that produce reactive oxygen species [73, 74]. The defense role of carotenoids against GLS in maize was confirmed by experiments of total carotenoid quantitation for the two maize genotypes in this study (Fig. 8c).

The use of molecular markers and MAS can facilitate gene stacking for more durable resistance, since multiple genes that are effective against the pathogens can be combined into a single breeding line or a variety in a manner that would not be possible with phenotypic selection only [75]. We have narrowed the potential protein candidates by co-localizing with previously mapped resistance QTL regions and screened the SNPs in each protein (Table 2). This method can promote the screening of potential resistance genes or molecular markers for GLS. In this study, the four proteins located in the resistance QTL regions (i.e., CML25, LRR-RLK, GST, and CCR3) were tightly associated with GLS resistance. QTL mapping of a maize recombinant inbred line (RIL) population derived from subtropical CML444 × SC Malawi maize inbred lines found that the QTL region in chromosome 6 (located in bin 6.06/6.07) explained more than 11% of phenotypic variation [22], CML25 and LRR-RLK, which also contains SNPs, were located in this region, indicating that they are most likely candidate resistance proteins. Additionally, SNP1-G, SNP2-A, and SNP4-A are potential molecular markers for MAS. However, the functions of these proteins or markers and their contribution to the enhancement of resistance to GLS need to be further verified. Moreover, GST (located in bin 6.04) and CCR3 (located in bin 6.01) mapped to other QTLs on chromosome 6 and are candidate proteins, because this region (qGLS6.01–6.04) was associated with 6.85% of variation in GLS resistance [20]. The remaining eight proteins were not located in any GLS-resistance QTL regions, which may have little effect on phenotypic variation. However, we failed to develop markers associated with these proteins by using RNA-Seq owing to the limited number of sampled populations; thus, further analysis could use genome-wide association prediction models to establish molecular markers associated with these proteins. Since we have developed markers for all relevant proteins, genomic selection is a better approach for the capture of little-effect genes than MAS. The reason for this is that the practical implementation of MAS for stacking multiple disease resistance genes is difficult; therefore, the population size needed for the maintenance and fixing of multiple genes quickly exceeds the reasonably available resources for MAS.

## Conclusions

To develop resistant cultivars and control GLS, understanding host–pathogen interactions is essential. In this study, novel GLS resistance genes and metabolites were identified by combining transcriptional, protein, and metabolic expression levels, and an interaction network of maize resistance to GLS was also constructed, which was regulated by ROS, SA, and Ca^2+^ signaling pathways as well as carotenoids. In particular, four genes on chromosome 6 that were consistently expressed at higher levels in the resistant cultivars and occurring within GLS resistance QTL regions seemed most promising, namely a CML25, a LRR-RLK, a GST, and a CCR3 protein. Further validation of the association of these proteins is necessary to improve our understanding of maize resistance to GLS. This information will provide new insights for molecular marker-assisted breeding of GLS-resistant maize cultivars.

## Additional files

Additional file 1: List of qPCR and PCR primers used in this study. (XLSX 12 kb)

Additional file 2: Evaluation of sequencing quality. Y and Z represent ‘Yayu889’ and ‘Zhenghong532,’ respectively; 1, 2, 3, and 4 represent 81, 89, 91, and 93 days after planting, respectively; a, b, and c represent each of the three biological replicates of each time point, respectively. (DOCX 17 kb)

Additional file 3: Mapping efficiency statistics. Y and Z represent ‘Yayu889’ and ‘Zhenghong532,’ respectively; 1, 2, 3, and 4 represent 81, 89, 91, and 93 days after planting, respectively; a, b, and c respectively represent each of the three biological replicates of each time point, respectively. (DOCX 19 kb)

Additional file 4: Correlation coefficients between each pair of three biological replicates. (XLSX 14 kb)

Additional file 5: Up-regulated and down-regulated chitinases in ‘Yayu889’ and ‘Zhenghong532’ at 89, 91, and 93 days post planting, respectively. (XLSX 15 kb)

Additional file 6: Up-regulated and down-regulated PR genes in ‘Yayu889’ and ‘Zhenghong532’ at 89, 91, and 93 days post planting, respectively. (XLSX 11 kb)

Additional file 7: Up-regulated and down-regulated wall-associated kinases in ‘Yayu889’ and ‘Zhenghong532’ at 89, 91, and 93 days post planting, respectively. (XLSX 16 kb)

Additional file 8: Up-regulated and down-regulated MAP kinases in ‘Yayu889’ and ‘Zhenghong532’ at 89, 91, and 93 days post planting, respectively. (XLSX 14 kb)

Additional file 9: Up-regulated and down-regulated WRKY transcription factors in ‘Yayu889’ and ‘Zhenghong532’ at 89, 91, and 93 days post planting, respectively. (XLSX 15 kb)

Additional file 10: Up-regulated and down-regulated glutathione *S*-transferase in ‘Yayu889’ and ‘Zhenghong532’ at 89, 91, and 93 days post planting, respectively. (XLSX 15 kb)

Additional file 11: List of differentially expressed genes (DEGs) involved in the ‘Plant-pathogen pathway’ in ‘Yayu889’ and ‘Zhenghong532’. (XLSX 28 kb)

Additional file 12: Top GO_BP of three co-expression modules in ‘Yayu889’. (XLSX 18 kb)

Additional file 13: List of 56 candidate resistance genes obtained by combining transcription profiling and co-expression network analysis. (XLSX 37 kb)

## Abbreviations

CML: Calmodulin-like
dap: Days post planting
DEGs: Differentially expressed genes
DG: Dark gray
DM: Dark magenta
GLS: Gray leaf spot
GO: Gene Ontology
GST: Glutathione-*S*-transferase
KEGG: Kyoto Encyclopedia of Genes and Genomes
LRR-RLK: Leucine-rich repeat receptor-like protein kinase
LYK: Lysin motif receptor kinase
MAPK: Mitogen-activated protein kinases
MAS: Markers-assisted selection
MKK: MAP kinase kinase
MRM: Multiple reaction monitoring
NILs: Near-isogenic lines
PAL: Phenylalanine ammonia-lyase
PAMP: Pathogen-associated molecular pattern
POD: Peroxidase
PU: Purple
QTL: Quantitative trait locus
ROS: Reactive oxygen species
SA: Salicylic acid
WAK: Wall associated kinases
WGCNA: Weighted Gene Co-expression Network Analysis
