# Supplementary material for: Transcriptome analysis reveals the molecular mechanisms of the defense response to gray leaf spot disease in maize
Source: BMC Genomics. 2018 Oct 11;19:742. doi: 10.1186/s12864-018-5072-4 (PMC6180411; doi:10.1186/s12864-018-5072-4)
Supplement: Supplementary file 2 — Evaluation of sequencing quality. Y and Z represent ‘Yayu889’ and ‘Zhenghong532,’ respectively; 1, 2, 3, and 4 represent 81, 89, 91, and 93 days after planting, respectively; a, b, and c represent each of the three biological replicates of each time point, respectively. (DOCX 17 kb) [file 12864_2018_5072_MOESM2_ESM.docx]

**Additional file 2** Evaluation of sequencing quality.

| **Sample ID** | **Obtained bases (G)** | **GC content (%)** | **% ≥ Q30** |
| --- | --- | --- | --- |
| **Y1-1** | 8.73 | 56.55 | 89.22 |
| **Y1-2** | 6.25 | 54.76 | 89.89 |
| **Y1-3** | 8.05 | 55.82 | 90.57 |
| **Y2-1** | 6.43 | 57.09 | 90.47 |
| **Y2-2** | 8.77 | 57.29 | 89.84 |
| **Y2-3** | 7.12 | 57.09 | 89.66 |
| **Y3-1** | 6.48 | 56.52 | 89.80 |
| **Y3-2** | 6.47 | 57.04 | 89.86 |
| **Y3-3** | 7.91 | 56.64 | 90.38 |
| **Y4-1** | 6.93 | 56.78 | 89.32 |
| **Y4-2** | 6.76 | 56.57 | 90.70 |
| **Y4-3** | 6.27 | 56.50 | 90.44 |
| **Z1-1** | 7.28 | 55.94 | 87.40 |
| **Z1-2** | 6.62 | 56.51 | 88.17 |
| **Z1-3** | 8.37 | 56.15 | 88.71 |
| **Z2-1** | 7.58 | 58.01 | 88.11 |
| **Z2-2** | 10.63 | 57.49 | 88.54 |
| **Z2-3** | 8.03 | 57.80 | 88.05 |
| **Z3-1** | 9.36 | 55.97 | 88.33 |
| **Z3-2** | 7.63 | 56.80 | 88.51 |
| **Z3-3** | 9.09 | 56.73 | 88.15 |
| **Z4-1** | 8.71 | 57.55 | 87.50 |
| **Z4-2** | 7.18 | 56.53 | 87.65 |
| **Z4-3** | 8.71 | 56.81 | 88.12 |

Y and Z, Yayu889 and Zhenghong532, respectively.

Number 1, 2, 3 and 4 represent the samples collected in 81, 89, 91 and 93 days post planting, respectively, three biological replicates were list as 1, 2 and 3.
