# Supplementary material for: Transcriptome analysis reveals the molecular mechanisms of the defense response to gray leaf spot disease in maize
Source: BMC Genomics. 2018 Oct 11;19:742. doi: 10.1186/s12864-018-5072-4 (PMC6180411; doi:10.1186/s12864-018-5072-4)
Supplement: Supplementary file 3 — Mapping efficiency statistics. Y and Z represent ‘Yayu889’ and ‘Zhenghong532,’ respectively; 1, 2, 3, and 4 represent 81, 89, 91, and 93 days after planting, respectively; a, b, and c respectively represent each of the three biological replicates of each time point, respectively. (DOCX 19 kb) [file 12864_2018_5072_MOESM3_ESM.docx]

**Additional file 3** Statistics of mapping efficiency

| **Sample** | **Total Reads** | **Mapped Reads**  **(%)** | **Uniq Mapped**  **Reads (%)** | **Multiple Map Reads (%)** | **Reads Map to**  **'+' (%)** | **Reads Map to**  **'-' (%)** |
| --- | --- | --- | --- | --- | --- | --- |
| **Y1-1** | 55,136,160 | 65.49 | 60.04 | 5.45 | 31.54 | 31.48 |
| **Y1-2** | 44,035,042 | 65.22 | 59.94 | 5.28 | 31.48 | 31.49 |
| **Y1-3** | 60,799,448 | 65.85 | 59.67 | 6.18 | 31.54 | 31.51 |
| **Y2-1** | 48,770,496 | 69.74 | 65.20 | 4.53 | 33.91 | 33.86 |
| **Y2-2** | 44,217,454 | 69.24 | 65.46 | 3.77 | 33.87 | 33.85 |
| **Y2-3** | 44,012,850 | 69.96 | 66.15 | 3.81 | 34.21 | 34.17 |
| **Y3-1** | 53,753,404 | 64.33 | 59.83 | 4.49 | 31.27 | 31.18 |
| **Y3-2** | 46,944,190 | 67.73 | 63.65 | 4.08 | 33.04 | 33.01 |
| **Y3-3** | 46,382,280 | 68.04 | 61.98 | 6.07 | 32.79 | 32.73 |
| **Y4-1** | 42,736,472 | 67.66 | 63.04 | 4.62 | 32.90 | 32.83 |
| **Y4-2** | 50,422,536 | 68.64 | 62.36 | 6.28 | 33.03 | 32.95 |
| **Y4-1** | 45,120,144 | 69.09 | 63.77 | 5.32 | 33.51 | 33.43 |
| **Z1-1** | 42,326,542 | 68.53 | 60.49 | 8.04 | 32.51 | 32.54 |
| **Z1-2** | 56,567,140 | 68.58 | 64.67 | 3.92 | 33.48 | 33.54 |
| **Z1-3** | 59,047,372 | 67.27 | 64.23 | 3.04 | 33.00 | 33.03 |
| **Z2-1** | 51,007,830 | 67.57 | 63.61 | 3.96 | 33.01 | 32.94 |
| **Z2-2** | 71,729,732 | 68.64 | 64.28 | 4.36 | 33.46 | 33.40 |
| **Z2-3** | 54,083,798 | 68.20 | 63.81 | 4.39 | 33.21 | 33.12 |
| **Z3-1** | 63,083,728 | 68.26 | 64.81 | 3.45 | 33.48 | 33.49 |
| **Z3-2** | 51,308,390 | 70.01 | 66.88 | 3.13 | 34.44 | 34.43 |
| **Z3-3** | 61,127,954 | 70.10 | 66.58 | 3.51 | 34.37 | 34.36 |
| **Z4-1** | 58,534,270 | 68.52 | 65.34 | 3.18 | 33.66 | 33.61 |
| **Z4-2** | 48,290,704 | 70.92 | 67.61 | 3.31 | 34.86 | 34.81 |
| **Z4-3** | 58,471,592 | 70.78 | 66.79 | 3.99 | 34.60 | 34.57 |

Y and Z, Yayu889 and Zhenghong532, respectively.

Number 1, 2, 3 and 4 represent the samples collected in 81, 89, 91 and 93 days post planting, respectively, three biological replicates were list as 1, 2 and 3.
